# Glans Preserving Buccal Mucosa Urethroplasty for Glandular and Distal Urethral Strictures

**DOI:** 10.5152/tud.2022.22024

**Published:** 2022-07-01

**Authors:** Christophe De Laet, Gunter De Win

**Affiliations:** 1 Department of Urology, Antwerp University Hospital, Antwerp, Belgium; 2 University of Antwerp Faculty of Medicine and Health Sciences/ASTARC, Antwerp, Belgium; 3 Adolescent and Reconstructive Urology, University College London Hospitals, London, UK

**Keywords:** Penile stricture, glans preservation, buccal mucosa, penile urethroplasty, graft

## Abstract

**Background:**

To describe a step-by-step approach for glans preserving urethroplasty with a dorsal inlay graft used for distal urethral strictures.

**Description of the Technique:**

The reconstruction was performed through a keyhole incision in the urethra. In this way, we achieve maximal exposure by a minimal incision and saving of the glans. After incision of the diseased dorsal urethral mucosa through the keyhole and the meatus, a buccal mucosa graft pull-through resulting in a dorsal inlay is done.

**Patient(s) and Methods:**

We treated 10 patients in different clinical settings with success by using the newly described technique below. We highlight and illustrate 1 case of a 34-year-circumcised male. Antegrade urethrogram showed a distal penile and fossa navicularis stricture with a total estimated length of 3.5 cm.

**Results:**

In this specific case the glans sparing approach had a surgical duration of 115 minutes. After 3 weeks the urinary catheter was removed. At 12 months, the patient reported no remaining urinary tract symptoms. Examination showed a fully healed lesion and an adequate uroflowmetry with a *Q*_max_ of 24 mL/s coming from 4 mL/s pre-operatively. In our 10-patient case series, all treated patients had complete resolution of their complaints, significant improvement in flow rates and excellent cosmetic results without complications.

**Conclusion:**

In selected cases, the described technique is feasible, safe, and effective with excellent functional outcomes and better cosmetic results especially due to the glans preservation.

## Introduction

The etiology of distal penile and fossa navicularis (FN) strictures is variable. The main causes are instrumental/iatrogenic, lichen sclerosus et atroficus (LSA), failed hypospadias reconstruction surgery, and idiopathy. Involvement of the fossa navicularis is seen in 18% of the patients. When distal urethral strictures involve the FN (distal urethral stricture (DUS)+FN), the repair entails not only achieving long-term urethral patency but also cosmetic results.^1,2^ The treatment of penile and distal urethral strictures including the FN and the meatus remains challenging. Direct vision internal urethrotomy is not advised for penile strictures and urethroplasty is associated with significantly better long-term success rates. Nowadays, the trend is to use buccal mucosa grafts over other penile fasciocutaneous skin flaps, ventral preputial skin island flaps, or other free grafts. The skin flaps might even be contra-indicated in the case of LSA. Men with (distal) penile urethral strictures can be offered a single-stage or staged approach taking into consideration previous interventions and stricture characteristics. However, the possible need to open the glans, lack of ventral tissue support, and adverse etiology often make it surgically difficult. Our aim with this technique is to contribute to better cosmetics of the glans and prevent dehiscence or fistula formation, 2 complications that are regularly seen.

## Patient and Methods

### Assessment

**Figure 1.**
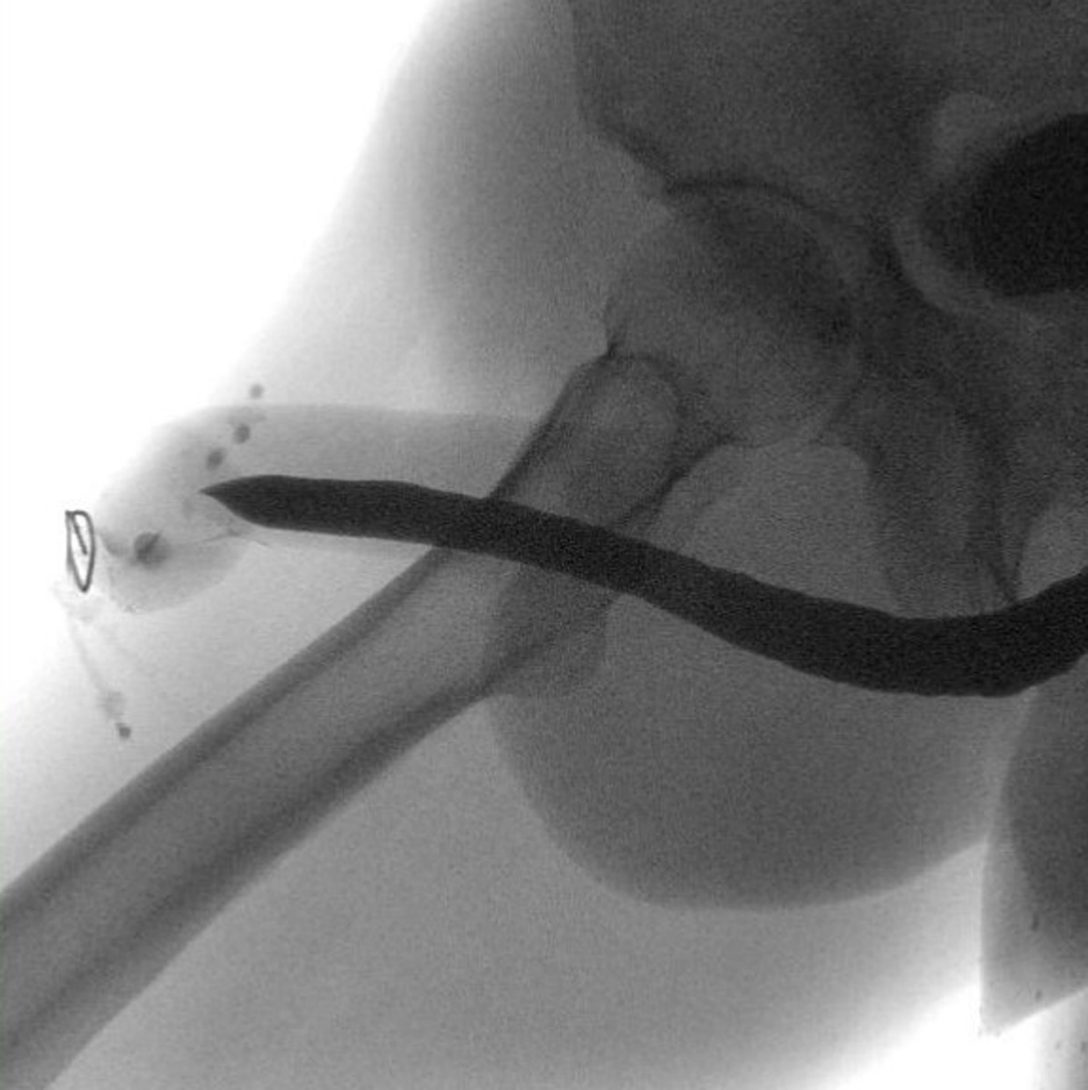
Antegrade urethrogram of the patient shows an obliterative stricture of approximately 3.5 cm starting from the subcoronal and running up to the fossa navicularis. Dilatation of the remaining anterior urethra is seen, running up to a triangular complete obliteration. The posterior urethra, although not in the picture, was free of any lesions.

A 34-year-old circumcised male presented with bothersome voiding lower urinary tract symptoms and urinary tract infection. Clinical examination showed an obliterated meatus and LSA with an obstructive uroflowmetry with a *Q*_max_ of 4 mL/s. After inserting 5 Fr suprapubic lines, an antegrade urethrogram with a marker at the meatus was performed. A 3.5-cm length FN + DUS was seen. Additionally, proximal strictures were excluded because of clear dilatation of the proximal urethra (Figure 1). Being fully informed, the patient consented for surgery and gave consent for the use of his clinical information and for taking and processing images for scientific purposes. Ethical approval is exempt. There is no information or data that causes physical or psychological harm to patients or damages the integrity of the patient. The urethroplasty techniques proposed, were to be definitely decided per-operatively based on our surgical findings but are ethical and not considered as (experimental) human subject research. Written informed consent was obtained from all participants who participated in this study.

### Materials and Methods—Statistics

The article type is descriptive. Age, stricture length, preoperative *Q*_max_ values, and postoperative *Q*_max_ values are collected and will be analyzed with descriptive statistics. Interpretation is done by using the Shapiro–Wilk test of normality. When normal distribution is confirmed a paired sample *t*-test will be performed to compare *Q*_max_ pre- and postoperatively. Data analysis is done using the Statistical Package for the Social Sciences version 28.

### Description of Technique

The surgical team consists of at least 3 persons including 1 surgeon, 1 assistant and 1 scrub nurse. If available, the second team of at least 2 members could simultaneously harvest and prepare the buccal mucosa graft thereby drastically reducing the operating time up to 35 minutes.

**Figure 2.**
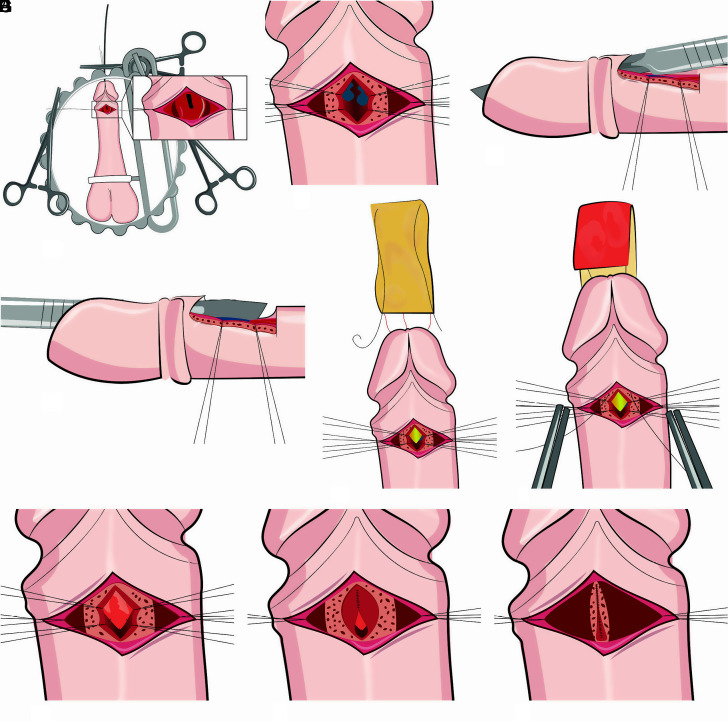
A schematic drawing of the key steps in the surgical process. (A) At the start, our positioning with the use of the traction suture, the Joshi-Kulkarni retractor, and the Dennis Brown ring is seen. Skin incision has been made and the probe is positioned adequately for keyhole incision in the marked urethra. The urethra is lying free from the surrounding tissue. (B) Exposed dorsal mucosal layer with a clear view of the transition from diseased to healthy tissue. Differentiation is enhanced by using methylene blue. (C and D) Dorsal urethral mucosa is incised in both directions (through the keyhole and through the meatus), starting from the transition region up to the glans penis. (E) Positioning of the graft with the mucosal side down and fixating it distally at the glans with 3 sutures. (F) Pull-through mechanism by which the mucosal side is positioned facing up conform a dorsal inlay procedure. (G) The graft is positioned adequately to cover all incised, diseased dorsal mucosa and fixated proximally onto healthy mucosa without traction on the graft. (H) Closure of the keyhole incision in the ventral mucosal layer of the urethra. (I) Closure of corpus spongiosum followed by the closing of the skin.

**Figure 3.**
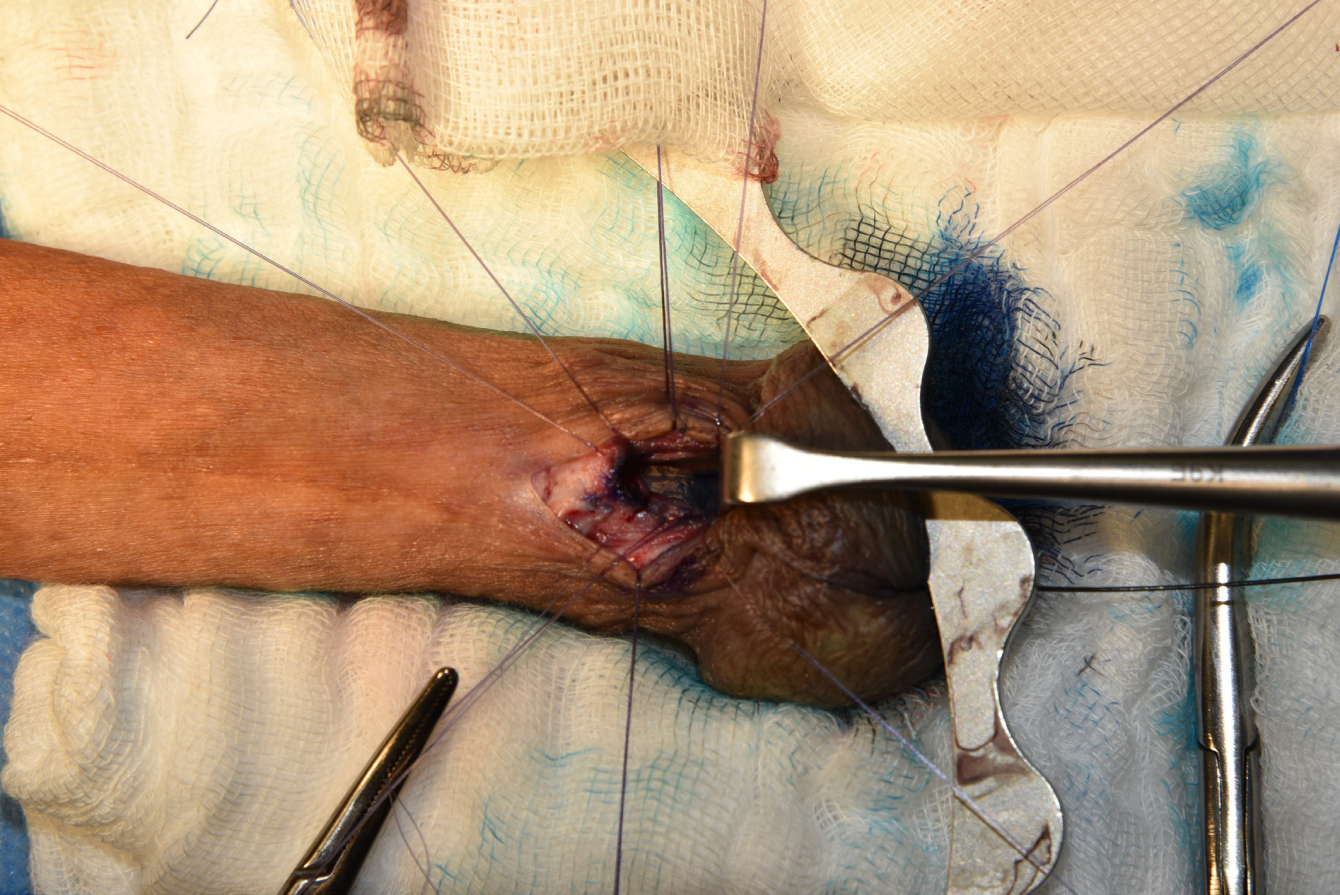
Overview of the keyhole incision with different traction sutures on the skin and ventral urethral mucosa. Through the opening, the healthy tissue can be clearly separated from diseased mucosa that colors with Methylene blue.

**Figure 4.**
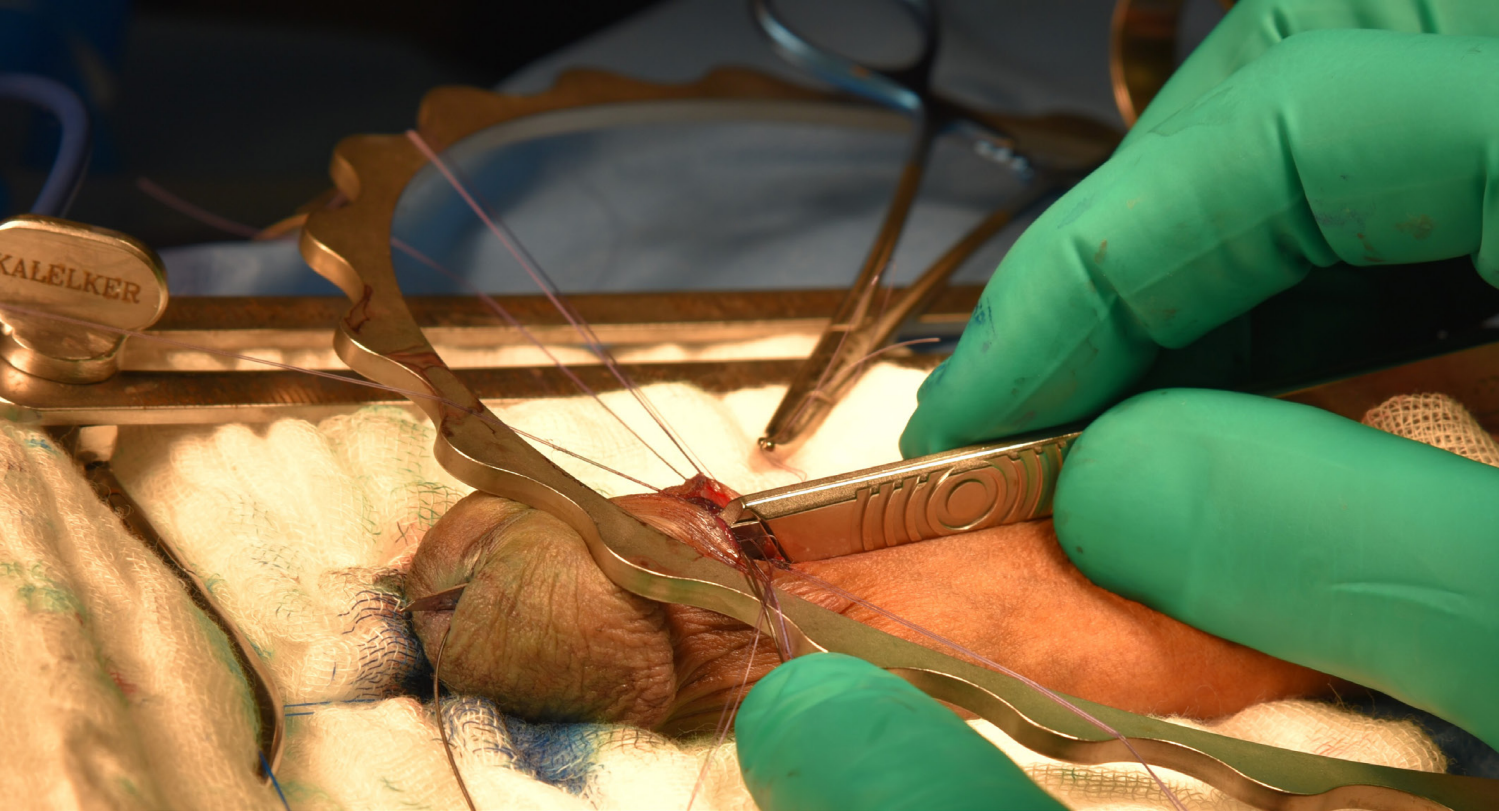
Incision of the dorsal urethra with N°11 scalpel through the keyhole incision. The same is done in the opposite direction from the meatus to the distal urethra. A Debakey forceps is used to spread the glans or the distal urethral opening to guide the incisions. The Joshi-Kulkarni retractor and Dennis Brown ring are used for optimal exposure.

**Figure 5.**
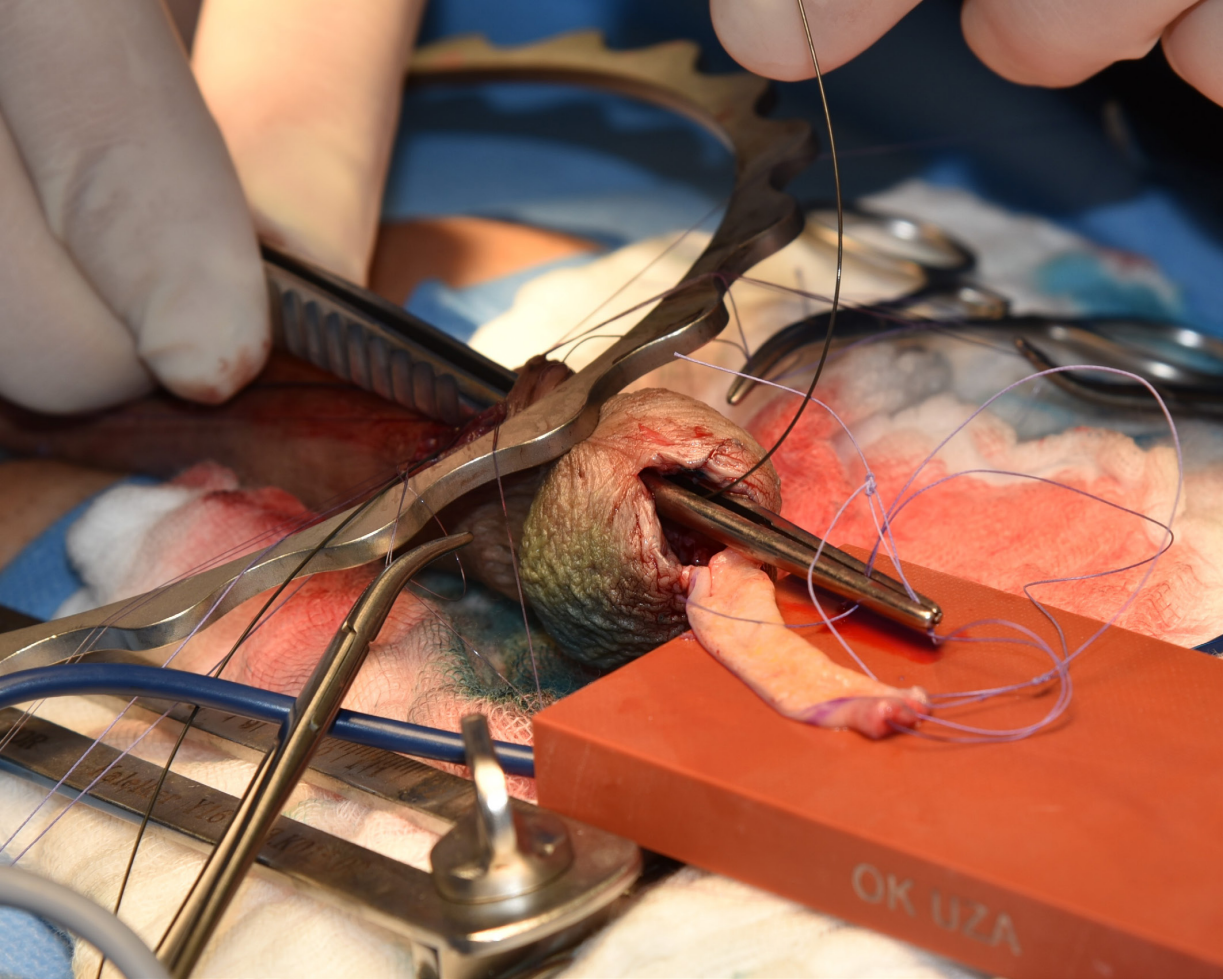
The graft is fully prepared and in this picture already been sutured to the glandular mucosa. It is placed with the mucosal side down and by performing the pull-through maneuver positioned correctly facing up, guided by the “marked” sutures.

**Figure 6.**
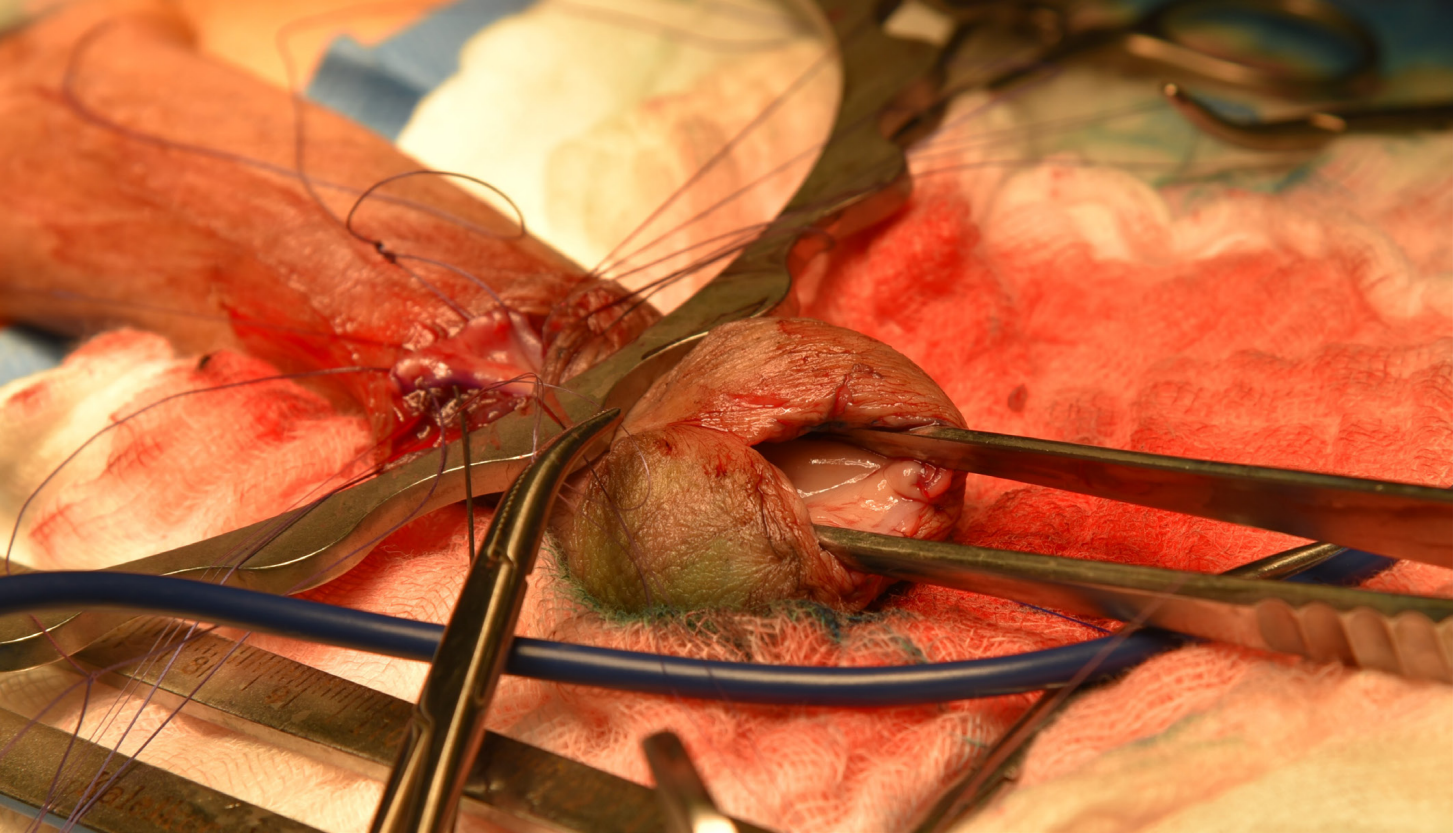
By introducing a forceps into the glans and then rotating it left and right, further fixation of the lateral edges and some quilting sutures can be performed much easier. Finally, complete attachment of the buccal mucosa graft at the distal part onto healthy mucosa is realized through the incision.

A broad-spectrum antibiotic, co-amoxiclav because of the mouth flora, is administered intravenously with general anesthesia. The patient is placed supine, disinfected, and draped sterilely. In all cases, a cystoscopy using a pediatric, ultrathin 4.5-6.5 Fr ureterorenoscopy with inspection of the penile urethra is performed in the operating room. The cystoscopy unit is placed at the feet of the patient. Additional information about the exact stricture location and stricture length is obtained and the location for skin incision is marked on the transition from diseased to healthy mucosa. Transition is distinguished with the help of methylene blue that is injected. Another relook inside the urethra for the exact location of the vertical keyhole incision is done after carefully dissecting up to the urethra. The operation is then started ideally by placing a traction suture through the glans (non-cutting 4/0 polypropylene suture) before the cystoscopy and installing the Joshi-Kulkarni retractor before incision (Figure 2).^3^ A small 1 cm horizontal skin incision on our marked spot is performed and 2 stay sutures are placed. Further dissection is done until the urethra is identified. We do a relook cystoscopy and this time mark the transition region on the urethra. Thereafter we incise the urethra over a slightly curved pediatric dilator or lacrimal probe that is placed through the meatus up to the level of our marking. This vertical, keyhole incision of the urethra is vital in preserving the glans (Figure 2). Different directions of subsequent incisions are used to prevent fistula formation after closure of the wound. More stay sutures through the spongiosum and the ventral mucosal layer are placed on either side. The ventral urethral wall is incised longitudinally with iris scissors for maximal exposure and visualization of the transition zone (Figure 3). The dorsal urethra is incised with a blade size 11 by inserting it through the keyhole opening until exposed in the meatal opening and then retracting the scalpel (Figures 2 and 4). A Debakey forceps is introduced to widen and maximally spread the meatus allowing easier incision of the dorsal urethra. A deep glans incision in the midline is followed by parallel incisions to widen the urethra. A buccal mucosa graft is harvested.^4,5^ The graft is first sutured to the most distal part of the glans penis at 3 different positions (left, right, and central) with a monofilament 5/0, if necessary the edges are trimmed to fit the curvature of the glans. The graft is positioned with the lingual side down. The proximal edge is marked with a short (right) and long (left) suture for positioning. By placing a Debakey forceps through the keyhole in the urethra it is maximally exposed, the sutures are grabbed and pulled through the glans under direct vision (Figures 2 and 5). Position is checked and the graft is fixated proximally onto dorsal healthy mucosa with monocryl sutures. We make sure that there is no traction on the graft as this could cause some pullback of the glans penis (Figure 2). By placing the forceps in the glans and rotating it 90°, then applying gentle pressure downwards and laterally, some quilting sutures are placed to fix the graft (Figure 6). **The proximal part of the graft is quilted through the key hole incision, the distal part through the meatus. The more proximal part of the graft inside the glans cannot be reached for quilting. The position and patency are checked and correct fixation of the dressing with the transurethral catheter on the abdomen at the end of the procedure is of utmost importance to keep the central part of the graft fixed in a dorsal position.** Finally, the lateral, ventral margins of the urethra are approximated and closed ventrally over a 16 Fr silicone catheter with a continuous inverting suture. Spongiosum is closed next to cover it, both with a monofilament 5/0 (Figure 2). The keyhole skin incision is closed with a running subcutaneous delayed absorbable suture. We apply a soft penile dressing before fixation of the penis and the catheter to the lower abdomen. This gently pushes down the graft and prevents catheter pull and friction.

If a circumcision is not yet performed and is necessary, it can be carried out without any problems. In that case, no skin marking is necessary at the start of the surgery but a small deglovement is performed instead of a superficial skin incision. The deeper layers of corpus spongiosum and ventral urethra are still incised vertically with a keyhole access. **In case of a near obliterate strictures with a narrow urethral plate, the glans incision has to be made fairly deep. If at this point a deep incision is not sufficient, a ventral graft with double faced procedure can be done with the use of the Nikolavsky technique.** ^6^

## Results

**Figure 7.**
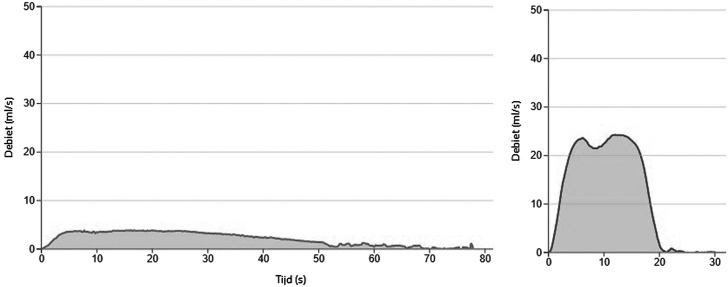
The pre- and postoperative uroflowmetry.

All procedures were completed in under 2 hours. The catheter was successfully removed in the outpatient clinic after 3 weeks. A peri-catheter urethrogram can be performed but is not done routinely. **A pre- and postoperative uroflowmetry shows clear improvement in the flow rate **(Figure 7). **In our dataset,** **all 4 parameter groups are found to be normally distributed. The mean age is 43 years old (standard deviation (SD) = 12). The mean stricture length is 30.8 mm (SD = 8.2 mm). The mean preoperative flow is 7.8 mL/s (SD = 3.5 mL/s) and the mean postoperative flow is 25.4 mL/s (SD = 6.8 mL/s). Paired samples test showed a significant flow rate improvement after urethroplasty (** ***P*** **< .001). The descriptive parameters in our small dataset are illustrative and show that multiple patients are experiencing the benefit from the underwent surgery.**

With this technique, we also treated a failed hypospadias repair with fistula formation due to a narrow distal urethra. The fistula was used as a keyhole entrance. A single-stage procedure with double-faced urethroplasty through the keyhole incision was successful as well. Ventral graft inlay was done with external sutures through the skin and additional inside-out quilting as described by Nikolavsky.^6^

In our case series of 10 patients, the mean age was 41.6 ± 10.9 years. The stricture length ranged from 2 cm to 4.7 cm (mean 3.2 cm). Mean *Q*_max_ increased from 7.7 mL/s ± 3.5 ml/s pre-operatively to a mean *Q*_max_ of 25.4 mL/s ± 6.4 mL/s. All of the patients reported subjective improvement during voiding with a mean *Q*_max_ preoperatively of 7.7 mL/s ± 3.5 mL/s and a mean *Q*_max_ after surgery of 25.4 mL/s ± 6.4 mL/s. Wilcoxson signed-rank test indicated a significant flow rate improvement (*P *= .006). A successful urethroplasty was seen in all 10 patients and was defined by the absence of any obstructive symptoms, good cosmetic results, and no need for consecutive interventions on short-term (1 year) follow-up. No fistula formation or glans dehiscence was seen.

## Discussion

Direct vision internal urethrotomy for penile strictures is not recommended. Many reconstructive options are available for DUS but currently, there is no gold standard. Malone meatoplasty or skin flap meatoplasty apply in certain cases. Dorsal techniques described by Asopa are used in open reconstruction with complex strictures but require incision and splitting of the glans, leading to higher unsatisfactory penile cosmetics.^7^ Lichen sclerosus et atroficus as a frequent cause of the disease limits the use of genital skin and promotes the use of buccal mucosa grafts.^1,8^ More transurethral approaches to urethroplasty are used in an effort to avoid skin incisions and minimize urethral dissection but are limited to short strictures.^9^ The risk of incomplete exposure of healthy proximal mucosa for good anastomoses exists. A technique with ventral wedge resection and external suture tying of a meatal buccal mucosa graft by Nikolavsky works glans sparing but is difficult for longer strictures.^6^ There is a shift toward an increased use of single-stage procedures even in patients with multiple previous interventions that led to fibrotic and atrophic scarring of the urethral plate.^7,8,10^ This is possible due to the advancements and refinements in operative techniques and often arises because patients are hesitant to accept staged treatment.^10^ In that case, our technique can be combined with Nikolavsky’s (ventral external suture tying) and will result in a double-face procedure. Avoidance of glans splitting prevents possible glans dehiscence and fistula formation and is cosmetically important. The advantage of the explained technique is that glans preserving results in better cosmetic results with equal functional improvement and good graft healing and adherence after being pulled through the meatus and positioned adequately with an anastomosis on healthy tissue.

## Conclusion

The glans preserving technique with keyhole incision and graft pull-through with dorsal buccal mucosa inlay graft is a viable method for the treatment of distal urethral strictures. The technique is less invasive, is applicable in different settings, and has better cosmetic results on top of excellent functional outcomes.
